# Efficacy and safety of adjuvant glucocorticoid therapy for *Pneumocystis* pneumonia (PCP) in patients positive for HIV: a systematic review and meta-analysis

**DOI:** 10.3389/fphar.2026.1916907

**Published:** 2026-10-28

**Authors:** Zhiyong Tang, Rui Li, Xiaoying Chen, Qiang Su, Bin Han, Ming Yang

**Affiliations:** 1 Beijing Anzhen Nanchong Hospital of Capital Medical University & Nanchong Central Hospital, Nanchong, China; 2 The Affiliated Hospital of North Sichuan Medical College, Nanchong, China; 3 Nanchong Key Laboratory of Individualized Drug Therapy, Nanchong, China; 4 Xiamen Humanity Hospital, Xiamen, China

**Keywords:** efficacy, glucocorticoids, pneumocystis pneumonia, safety, systematic review

## Abstract

**Objective:**

To systematically evaluate the efficacy and safety of glucocorticoids as adjuvant therapy for Pneumocystis pneumonia (PCP) in patients positive for human immunodeficiency virus (HIV).

**Methods:**

Clinical randomized controlled trials or clinical controlled trials evaluating the efficacy and safety of glucocorticoids as adjuvant therapy for PCP in patients who are HIV-positive were retrieved from PubMed, Embase, Web of Science, and the Cochrane Library. Data extraction and methodological quality assessment were performed for eligible studies. Meta-analysis and systematic evaluation were conducted using RevMan v.5.1 and STATA v.12.0 software.

**Results:**

Nine articles (13 studies) were included. Compared with the controls, patients treated with glucocorticoids had significantly lower mortality rates (OR = 0.41, 95% CI [0.28–0.59], P < 0.00001), early deterioration rates (OR = 0.08, 95% CI [0.02–0.39], P = 0.002), and lower mechanical ventilation rates (OR = 0.32, 95% CI [0.19–0.55], P < 0.0001). Regarding safety, the incidence of adverse effects did not differ significantly between the two groups (OR = 0.74, 95% CI [0.33–1.70], P = 0.48).

**Conclusion:**

Adjuvant glucocorticoid therapy significantly reduces mortality, early deterioration, and mechanical ventilation rates in patients who are HIV-positive with PCP without increasing adverse reactions. Glucocorticoids may serve as a viable adjuvant treatment option for PCP in patients who are HIV-positive.

**Systematic Review Registration:**

https://www.crd.york.ac.uk/PROSPERO/home, identifier CRD420261418795.

## 1 Introduction

*Pneumocystis* pneumonia (PCP) is the most common opportunistic infection among individuals infected with human immunodeficiency virus (HIV) infection (Ahmadpour et al., 2024). Prior to the widespread availability of antiretroviral therapy (ART) and PCP prophylaxis, 70%–80% of patients with acquired immunodeficiency syndrome (AIDS) developed PCP, with an associated mortality of approximately 20%–40% (Hay et al., 1988). Although the incidence of PCP has declined markedly in industrialized countries following the introduction of ART, it remains a major global health concern. PCP is estimated to cause >400,000 cases annually worldwide (McDonald et al., 2021), and a recent global systematic review estimated the pooled prevalence of HIV-associated pneumocystosis at 35.4% (Ahmadpour et al., 2024). Furthermore, the disease burden continues to be substantial in low- and middle-income regions (Ahmadpour et al., 2024; McDonald et al., 2024; Wills et al., 2021). Severe PCP can trigger diffuse alveolar damage, impair gas exchange, and lead to respiratory failure, contributing to a high mortality rate. PCP has become a leading cause of hospitalization and death among patients infected with HIV globally (Yang et al., 2018).

Glucocorticoids, as classic anti-inflammatory and immunomodulatory agents, are widely used in the treatment of various inflammatory and immune-related disorders. For patients who are HIV-positive with moderate-to-severe PCP, adjunctive glucocorticoid therapy can significantly improve clinical outcomes, reduce mortality, and enhance survival rates (Maschmeyer et al., 2016). However, the current recommendations mainly rely on earlier randomized controlled trials (RCTs) and evidence-based medical studies (Bozzette et al., 1990; Gagnon et al., 1990; Walmsley et al., 1995; Briel et al., 2006; Briel et al., 2005; Ewald et al., 2015), with insufficient inclusion of recently published studies from the past decade (Newberry et al., 2017; Mundo et al., 2020). The application of outdated drug exposure–effect relationships to contemporary patients may compromise both efficacy and safety, posing a major barrier to precise clinical medication. In addition, the immunosuppressive effect induced by glucocorticoids may weaken the ability of the host to clear pathogens. Patients with AIDS, in particular, are inherently immunodeficient. When such patients have PCP infection, the use of glucocorticoids can aggravate the infection. Therefore, whether to use glucocorticoids to treat this type of disease in clinical practice needs to be carefully re-evaluated.

The incorporation of recent clinical data is imperative and evidence-based medical analyses should be conducted to evaluate the efficacy and safety of adjunctive glucocorticoids in HIV-associated PCP treatment, with the aim to provide guidance for clinical practice.

## 2 Methods

The study protocol and analysis were performed in accordance with the Preferred Reporting Items for Systematic Reviews and Meta-Analyses (PRISMA) guidelines. The methodology applied was reported by Li et al. (2015).

### 2.1 Search strategy

A systematic search was conducted using Boolean logic across databases, including PubMed, Embase, Web of Science, and the Cochrane Library, to gather literature research on the efficacy and safety of using glucocorticoids as adjuvant therapy for PCP in patients who are HIV-positive. The search strategy included the following medical index terms: “Pneumocystis Pneumonia,” “Pneumocystis Infections,” “Pneumocystis carinii,” “Pneumocystis jirovecii,” “glucocorticoid,” “corticosteroid,” “steroid,” “HIV,” “AIDS,” “acquired immunodeficiency syndrome,” and “human immunodeficiency virus.” The search covered the period from the inception of each database to 10 December 2025. The references in the initially identified articles, including relevant reviews, were manually searched and reviewed to ensure that no relevant study was missed.

### 2.2 Study selection

Studies were included if they met the following inclusion criteria: (1) study types were RCT or clinical controlled trial (CCT); (2) the experimental group used agents containing glucocorticoids, whereas the control group either did not use glucocorticoids or received a placebo; (3) patients were HIV-positive with concurrent PCP; and (4) outcome measures included mortality rate, early deterioration rate, mechanical ventilation rate, and incidence of adverse effects (AEs). Studies were excluded if they had unavailable data, case reports, animal studies, or were duplicate studies.

All retrieved studies were scanned by two reviewers (ZYT and RL), who independently assessed all potentially relevant studies and then reached a consensus. In the case of disagreement between the two reviewers, the senior coauthor (MY) was consulted, and the disagreement was resolved by consensus (Li et al., 2018).

### 2.3 Data extraction

Two reviewers (ZYT and RL) extracted relevant data from the included literature studies independently. A predefined form was used to record the information, including: first author; publication year; patient age; follow-up duration; treatment regimen; mortality rate; early deterioration rate; incidence rate of AEs; mechanical ventilation rate; trial groups; evidence quality; allocation concealment; blinding implementation; completeness of outcomes; selective reporting; patients lost to follow-up and withdrawals.

### 2.4 Quality assessment

Two reviewers (ZYT and RL) independently assessed the methodological quality of all included studies without blinding with regard to the source journal or authorship. Disagreements were resolved by discussion or consultation with the third reviewer (MY) if required. Moreover, the risk of bias of the included randomized trials was further assessed using the revised Cochrane Risk of Bias tool (RoB 2), which covers five domains: bias arising from the randomization process, bias due to deviations from intended interventions, bias due to missing outcome data, bias in measurement of the outcome, and bias in selection of the reported result (Sterne et al., 2019). The overall certainty of the pooled evidence for each outcome was evaluated using the Grading of Recommendations Assessment, Development and Evaluation (GRADE) framework (Guyatt et al., 2008).

### 2.5 Data analysis and statistical methods

Statistical analysis was performed using Review Manager software (RevMan, v.5.1, Oxford, United Kingdom) and STATA software (STATA, v.12.0, StataCorp, College Station, TX, United States). Measurements and enumeration data were expressed as the mean difference and odds ratio (OR), respectively, with their 95% confidence intervals (CI). Heterogeneity among studies was assessed using the *P*-value and *I* ^2^ statistic. If *I* ^2^ > 50% and *P* < 0.05, significant heterogeneity was assumed, and a random-effects model was applied. Otherwise, the heterogeneity is small; a fixed-effects model (FEM) was used (Higgins et al., 2011). Sensitivity analysis was conducted to explore potential sources of heterogeneity. Publication bias was evaluated using Begg’s funnel plot, where asymmetry in the plot suggested potential bias (Li et al., 2018). Meanwhile, the Egger’s test was employed to statistically validate the presence of bias (Sterne et al., 2001), with *P* < 0.05 considered statistically significant.

## 3 Results

### 3.1 Literature search

The detailed literature search and article screening process is described in Figure 1. The complete search strategy used for each database is described in Supplementary Table S1. Through database searches, a total of 1,712 research articles were retrieved. After reviewing titles, abstracts, and full texts according to the inclusion and exclusion criteria, nine articles (Mundo et al., 2020; Montaner et al., 1989; Walmsley et al., 1995; Montaner et al., 1993; Bozzette et al., 1990; Alta et al., 2008; Gagnon et al., 1990; Montaner et al., 1990; Newberry et al., 2017) encompassing 13 studies were ultimately included in the meta-analysis.Of these, seven were single-study articles, whereas two were multi-study articles (one reporting two studies (Montaner et al., 2017) and another reporting four studies (Mundo et al., 2020).

**FIGURE 1:**
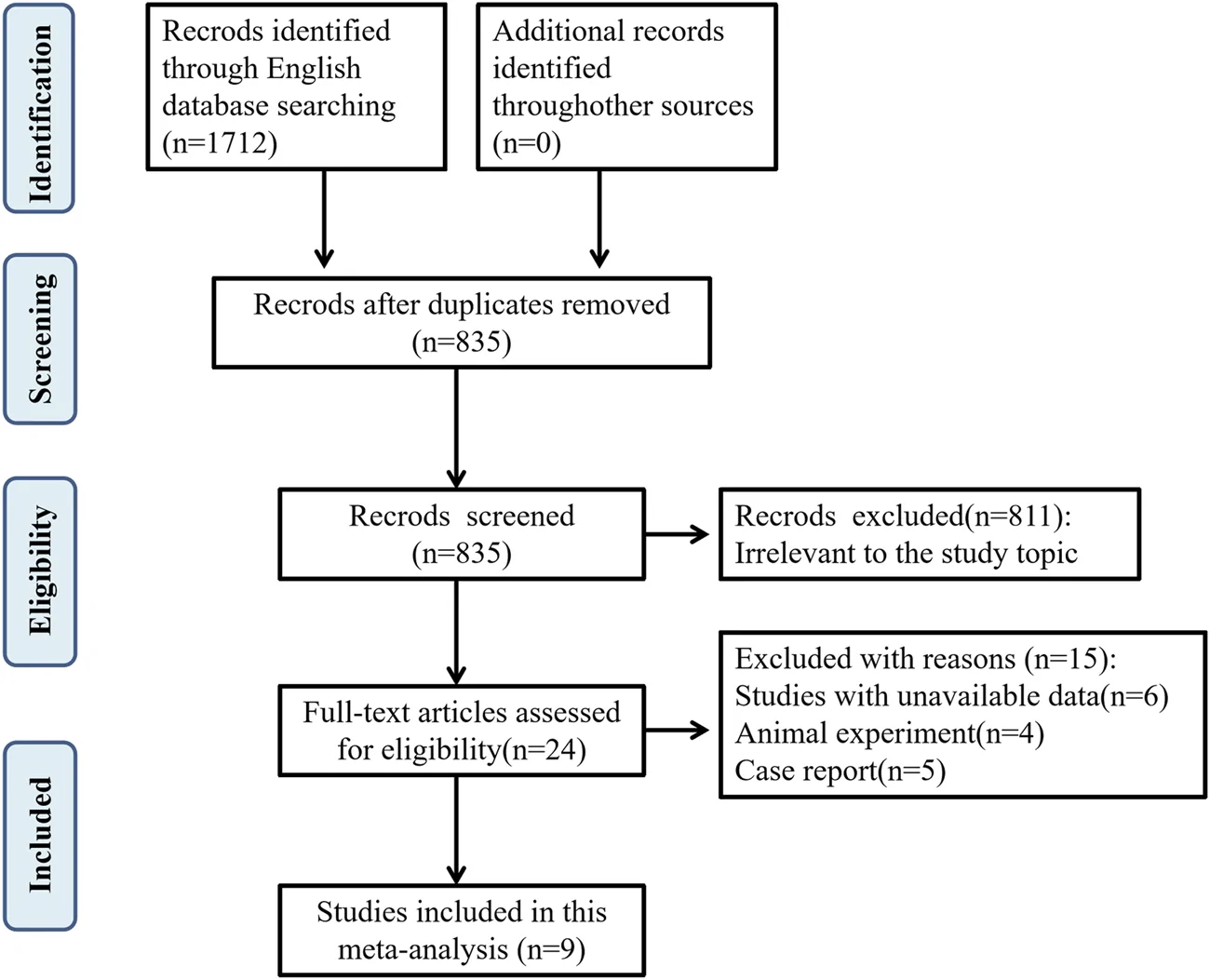
Flow diagram of studies included in the meta-analysis.

### 3.2 Study quality assessment and risk of bias assessment

The individual bias risk assessment for each included study is presented in Table 1. The bias risk results indicated that six studies demonstrated a low risk of bias (Walmsley et al., 1995; Montaner et al., 1993; Alta et al., 2008; Gagnon et al., 1990; Montaner et al., 1990; Newberry et al., 2017), whereas the remaining three studies showed some concern for bias risk (Mundo et al., 2020; Montaner et al., 1989; Bozzette et al., 1990). The risk of bias summary is shown in Supplementary Figure S1, with individual study assessments using the RoB two tool detailed in Supplementary Table S2. According to the GRADE framework, the certainty of the pooled evidence was rated as high for mortality and mechanical ventilation, given the large sample sizes and consistent findings across multiple studies. In contrast, the certainty was moderate for early deterioration and AEs, mainly because of the limited number of studies and small sample sizes available for these latter outcomes (Supplementary Table S6).

**TABLE 1:** Main characteristics of the studies included in the meta-analysis.

Study	Research type	Mean age	Treatment duration /Follow-up (days)	Enrolled patients (T/C)	Drug regimen		Assessment index	Risk of bias
					T	C		
Mundo 2020	CCT	38–75	Unclear	37/6	SMZ/TMP + GCs	SMZ/TMP	(1)(3)	Some concerns
Montaner 1989	CCT	24–44	Unclear	18/6	ST + HC	ST	(1)	Some concerns
Bozzette 1990	RCT	28–45	21/84	123/128	ST+ PNS 80 mg/d	ST	(1)(3)	Some concerns
Sharon1995	RCT	>18	21/180	40/38	SMZ/TMP+ MPS 40 mg/d	SMZ/TMP+ PBO	(1)(3)(4)	Low risk
Montaner 1993	RCT	23–61	14/30	11/12	SMZ/TMP+ PNS 60 mg/d	SMZ/TMP+ PBO	(1)(2)	Low risk
Alta 2008	RCT	Unclear	7/Unclear	47/31	SMZ/TMP+ PNS 2 mg/kg/d	SMZ/TMP+ PBO	(1)	Low risk
Gagnon 1990	RCT	23–66	7/Unclear	12/11	SMZ/TMP+ MPS 40 mg/d	SMZ/TMP+ PBO	(1)(3)	Low risk
Montaner 1990	RCT	Unclear	21/30	18/19	SMZ/TMP + PNS 60 mg/d	SMZ/TMP+ PBO	(2)	Low risk
Newberry 2017	RCT	<18	21/180	36/42	SMZ/TMP + PSL 2 mg/kg/d	SMZ/TMP+ PBO	(1)(4)	Low risk

Abbreviations: SMZ: sulfamethoxazole; TMP: trimethoprim; T: test group; C: control group; GCs: Glucocorticoids; HC: hydrocortisone; MPS: methylprednisolone; PNS: prednisone; PSL: prednisolone; PBO: placebo; CCT: Case-Control Trial; RCT: randomized controlled trial; ST: Standard Therapy. (1): Mortality rate; (2): Early deterioration rate; (3): Mechanical ventilation rate; (4): Adverse events.

### 3.3 Efficacy outcomes

#### 3.3.1 Mortality rate

A total of eight articles encompassing 11 studies reported mortality outcomes (Mundo et al., 2020; Montaner et al., 1989; Walmsley et al., 1995; Montaner et al., 1993; Bozzette et al., 1990; Alta et al., 2008; Gagnon et al., 1990; Newberry et al., 2017). The experimental group (receiving glucocorticoid therapy) comprised 431 patients, whereas the control group included 326 patients. Based on differing mortality assessment periods, the studies were stratified into three subgroups for analysis: assessment period ≤35 days, <35 days to ≤1 year, and unclear assessment period. Meta-analysis indicated low heterogeneity among the studies (*I* ^*2*^ = 34%, *P* = 0.13), and a FEM was employed for statistical analysis. Compared with the control group, the experimental group showed significantly lower mortality rates with (OR = 0.41, 95% CI [0.28–0.59], *P* < 0.00001) (Figure 2).

**FIGURE 2:**
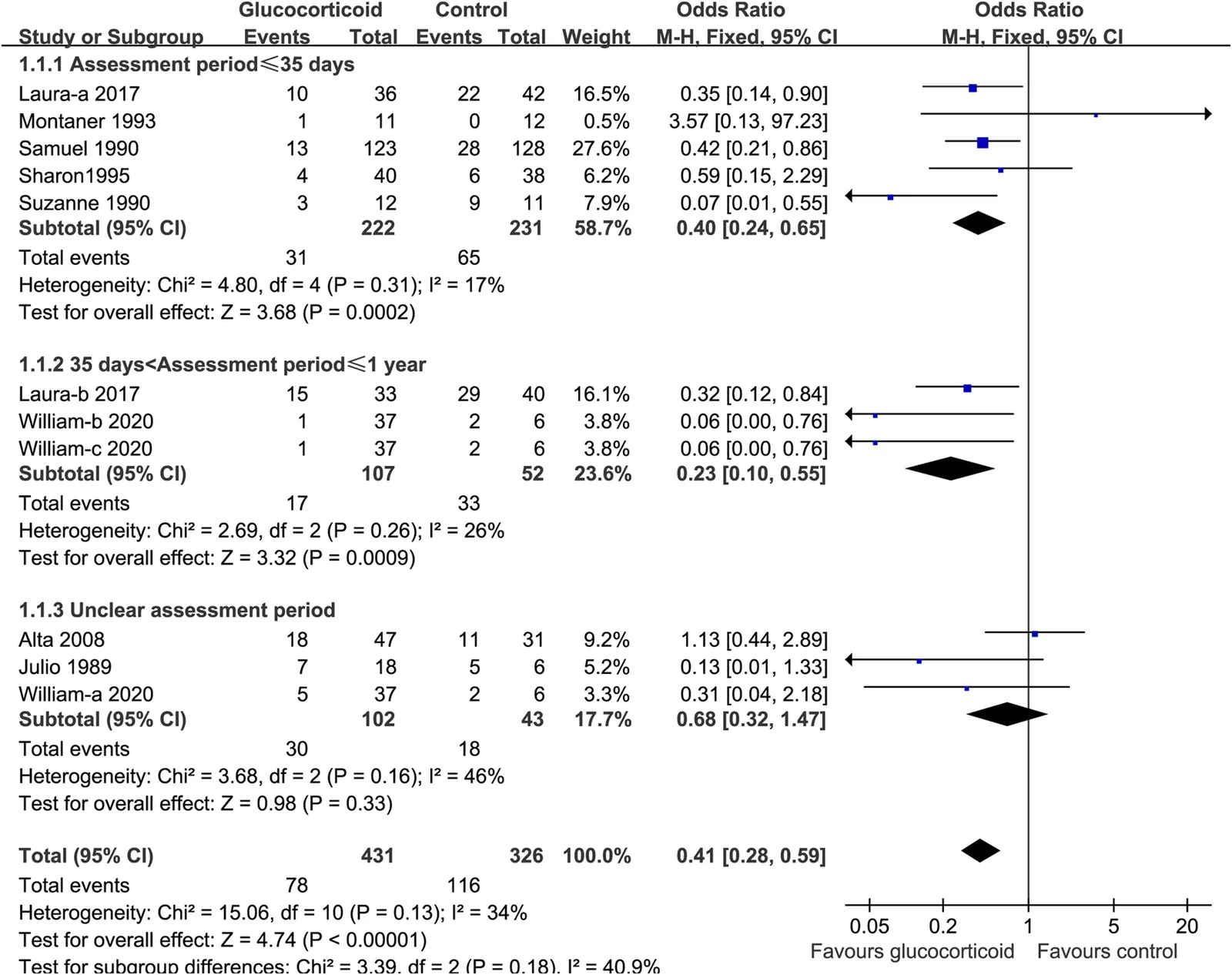
Forest plot for the comparison of the rate of mortality between the glucocorticoid group and the control group.

#### 3.3.2 Early deterioration rate

Among the nine included studies, two reported early deterioration rates (Montaner et al., 1993; Montaner et al., 1990). As shown in Figure 3, the experimental group (receiving glucocorticoid therapy) included 29 patients, while 31 patients were enrolled in the control group. Meta-analysis revealed no significant heterogeneity across studies (*I* ^*2*^ = 0%, *P* = 0.94), prompting the adoption of a fixed-effects model (FEM) for statistical analysis. Results demonstrated that the experimental group had significantly lower early deterioration rates compared to the control group (OR = 0.08, 95% CI [0.02–0.39], *P* = 0.002).

**FIGURE 3:**
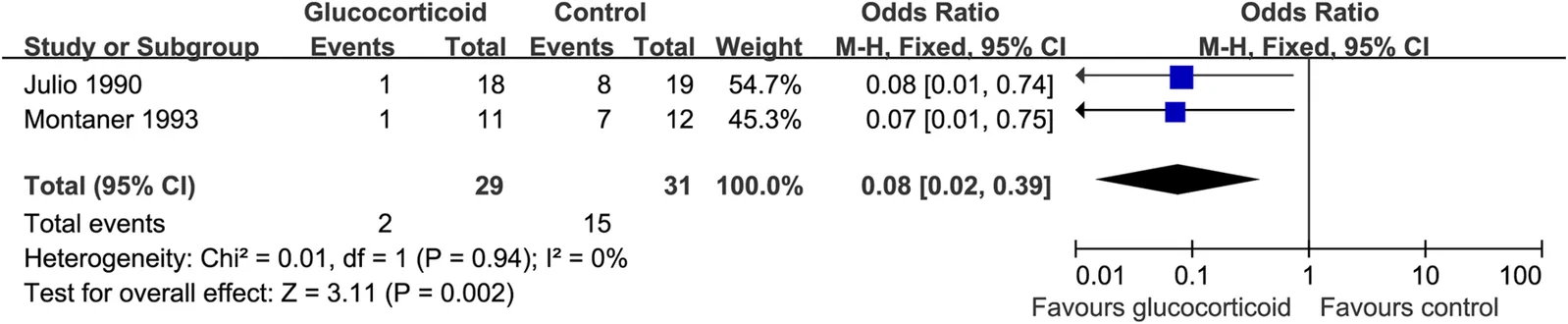
Forest plot for the comparison of the rate of early deterioration between the glucocorticoid group and the control group.

#### 3.3.3 Mechanical ventilation rate

Four studies investigated mechanical ventilation rates (Mundo et al., 2020; Walmsley et al., 1995; Bozzette et al., 1990; Gagnon et al., 1990), 212 patients were enrolled in the experimental group (receiving glucocorticoid therapy), and 183 patients were included in the control group (Figure 4). Meta-analysis revealed low heterogeneity among studies (*I* ^*2*^ = 43%, *P* = 0.16), therefore, a FEM was adopted for statistical analysis. Compared with the control group, the experimental group demonstrated a significantly lower mechanical ventilation rate with statistical significance (OR = 0.32, 95% CI [0.19–0.55], *P* < 0.0001).

**FIGURE 4:**
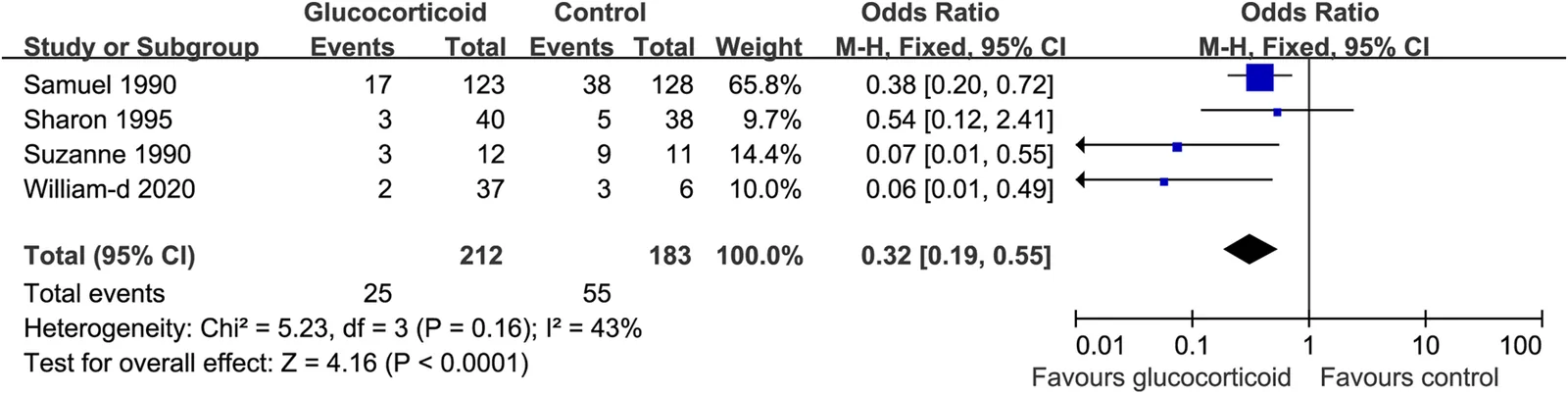
Forest plot for the comparison of the rate of mechanical ventilation between the glucocorticoid and control groups.

### 3.4 Safety outcomes

Two studies investigated the rate of AEs (Walmsley et al., 1995; Newberry et al., 2017), which involved the following organ systems: hematological system, digestive system, skin and appendages, and the central nervous system. As shown in Figure 5, the cases were categorized into these four subgroups based on AE types for analysis. The experimental group (receiving glucocorticoid therapy) included 196 patients, whereas the control group comprised 194 patients. The meta-analysis revealed low heterogeneity among the studies (*I* ^*2*^ = 29%, *P* = 0.23). Consequently, a FEM was employed for statistical analysis. The results demonstrated no significant difference in the AE incidence rate between the experimental and control groups (OR = 0.74, 95% CI [0.33–1.70], *P* = 0.48).

**FIGURE 5:**
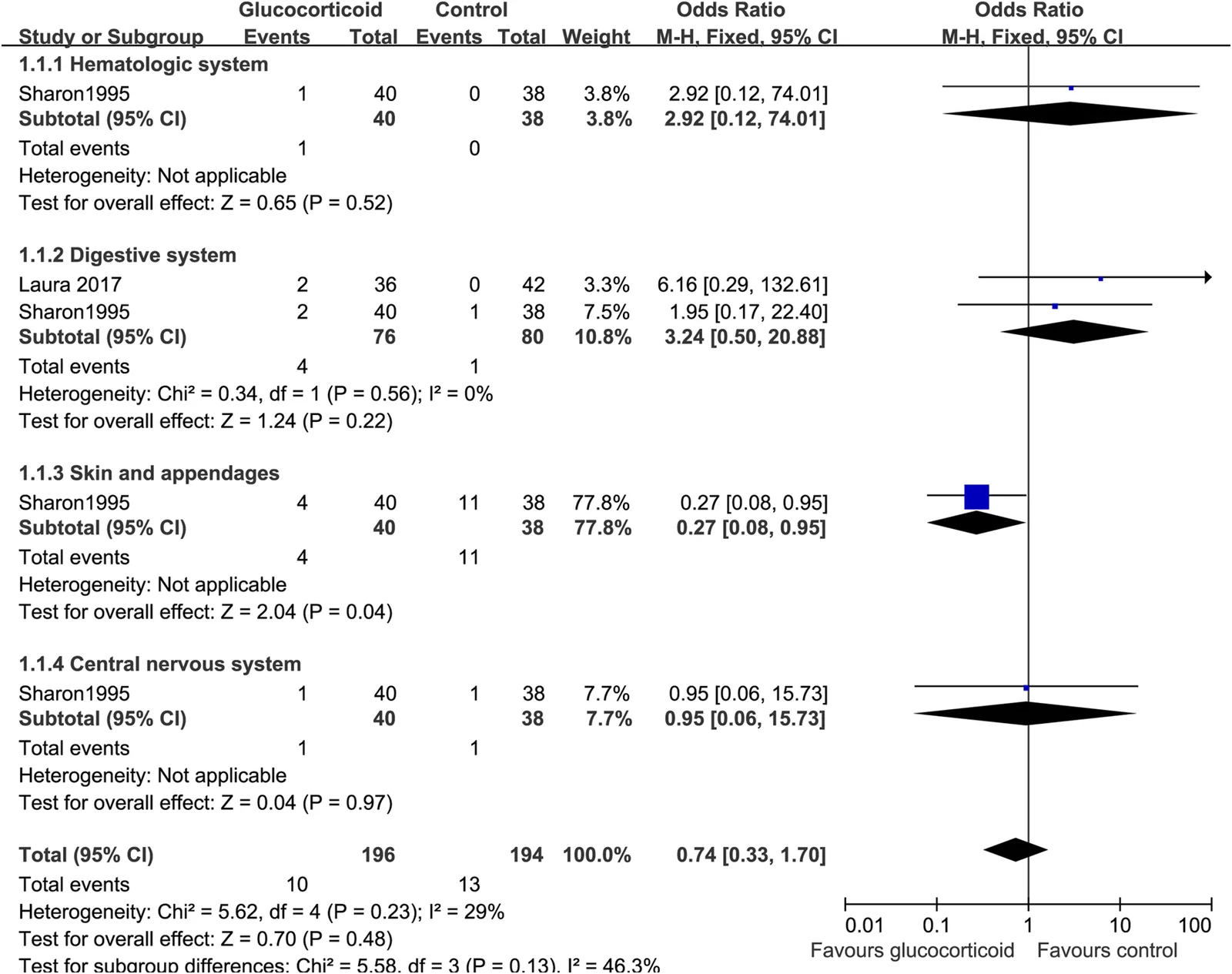
Forest plot for the comparison of the rate of AEs between the glucocorticoid group and the control group.

### 3.5 Publication bias analyses

Due to the limited number of studies reporting early deterioration rates and mechanical ventilation rates that could be included in the analysis, mortality data with larger sample sizes were selected for the analysis of publication bias. Begg’s funnel plot was generated using STATA v.12.0 software, where each circle represents an independent study (Li et al., 2023). As shown in Supplementary Figure S2, except for a few studies that lie outside the funnel plot due to higher heterogeneity, the circles in the funnel plot are generally symmetrically distributed. Egger’s test was further performed on the Begg’s funnel plot. The results showed a P-value of 0.192, confirming the absence of publication bias in this study. The funnel plot and the Egger’s test results are provided in Supplementary Figure S2 and Supplementary Table S3, respectively. Nevertheless, given the limited number of studies available for this analysis, the statistical power to detect small-study effects is low, and these results should be interpreted with caution.

### 3.6 Sensitivity analyses

A sensitivity analysis was performed by sequentially excluding studies with larger sample sizes on mortality. By removing each included study one at a time, the results indicated that the primary source of heterogeneity among the studies originated from Alta et al. (2008) (Supplementary Table S4). After removing this study, heterogeneity among the remaining studies significantly decreased (*I* ^2^ = 6%, *P* = 0.39). A FEM was applied for meta-analysis, and the results revealed that the comparison of mortality rates between the two groups still demonstrated a statistically significant difference (OR = 0.34, 95% CI [0.22–0.51], *P* < 0.00001).

Additionally, sensitivity subgroup analyses were conducted to explore potential sources of heterogeneity, including variations in follow-up duration (clearly defined and unclear), type of glucocorticoids (unknown, prednisone, prednisolone, hydrocortisone, or methylprednisolone), dose of glucocorticoids (unknown, methylprednisolone 40 mg/d, prednisone or prednisolone 2 mg/kg/d, prednisone 60 mg/d, or prednisone 80 mg/d), treatment duration (unknown, 7 days, 14 days, and 21 days), and patient population (adults and children). The results demonstrated consistent conclusions across all outcome measures; based on *P*-values and *I* ^*2*^ statistics, no significant heterogeneity was observed for outcomes other than the rate of AEs (Supplementary Table S5). However, after subgroup analyses of the AEs according to the different sources of heterogeneity mentioned above, heterogeneity increased (*I* ^*2*^ = 29% vs. *I* ^*2*^ = 59%), which was primarily attributed to the reduced total sample size of patients in both the glucocorticoid and control groups (subgroup analysis by AE types would expand the sample size).

## 4 Discussion

This study verified the efficacy and safety of glucocorticoid adjunctive therapy in patients who are HIV-positive with PCP from an evidence-based medicine perspective.

Regarding efficacy, the analysis revealed that HIV-positive patients with PCP receiving glucocorticoids showed significant benefits in reducing mortality rates, early deterioration rates, and mechanical ventilation rates, consistent with findings from studies by Wang et al. (2016) and Ewald et al. (2015). Glucocorticoids, renowned for their potent anti-inflammatory properties, effectively suppress inflammatory responses triggered by physical, chemical, immune, or pathogenic factors. In PCP treatment, glucocorticoids alleviate inflammatory reactions caused by the lysis of *Pneumocystis jirovecii* in the lungs, thereby improving oxygenation (Fishman et al., 2019). This mechanism explains the reduced mechanical ventilation rates and mortality observed in glucocorticoid-treated patients. It is worth noting that two studies provided survival rate data (Alta et al., 2008; Gagnon et al., 1990), although the data comprised the total number of people using glucocorticoids minus the number of deaths. Moreover, this meta-analysis study included the above two articles when analyzing the mortality rate, and the survival rate data contained in the two articles were relatively small. To reduce the possibility that a small sample size might mask potential survival benefits, survival rate outcome was not assessed as an indicator.

Regarding safety, the present analysis indicated no significant difference in overall AE incidence rates between the glucocorticoid and the control groups. However, the glucocorticoid group exhibited a higher incidence of severe AEs, particularly in the hematological (2.50% vs. 0%) and digestive systems (5.26% vs. 1.25%). In contrast, skin and appendage-related AEs were less frequent in the glucocorticoid group (10.0% vs. 28.9%). The reason for this is that the experimental groups extracted from the literature used glucocorticoids as part of the standard treatment plan. Glucocorticoids themselves have a higher risk of associated adverse drug redaction (ADRs), but they also exhibit anti-allergic effects (Ning, 2023). Allergic reactions (such as rashes) that patients are prone to self-discover and identify are easily masked. Therefore, the incidence of ADR of the skin and its appendages in the glucocorticoid group was less than that in the control group. Consequently, clinicians must vigilantly monitor and actively manage glucocorticoid-associated AEs in HIV-positive PCP patients to prevent prolonged disease courses and compromised prognoses. It should be emphasized, however, that only two studies contributed to the safety analysis, with limited sample sizes and few reported events; the statistical power to detect rare but clinically important harms (e.g., hyperglycemia, secondary infections, gastrointestinal bleeding, and herpesvirus reactivation) is therefore insufficient. Consequently, the absence of a statistically significant difference should not be interpreted as proof of safety equivalence, because small samples in safety analyses may underestimate uncommon but serious harms. Close monitoring for glucocorticoid-associated toxicity remains warranted in clinical practice.

Sensitivity analysis revealed that upon sequentially excluding each included study and re-performing the meta-analysis, heterogeneity primarily originated from the Alta et al. study (Alta et al., 2008). The clinical trial methodology employed in this study and included population (infants <18 months of age) may have contributed to its substantial heterogeneity. Subgroup analyses stratified by follow-up duration, glucocorticoid type, dose, treatment duration, and patient population for each outcome yielded consistent results (Supplementary Table S5), further confirming the robustness of the main findings. Except for the rate of AEs, for which the increased heterogeneity was primarily attributed to the relatively limited sample size, the statistical heterogeneity across the remaining outcomes was low to moderate (I^2^<50%), although the clinical heterogeneity among the included studies should still be acknowledged. The included trials spanned more than 3 decades (1989–2020) and varied in glucocorticoid type, dose, treatment duration, and follow-up duration. From the perspective of the primary outcome measure, mortality, and sensitivity subgroup analyses showed that the glucocorticoids associated with lower mortality in the glucocorticoid group were prednisone (17%) and methylprednisolone (13%). The lowest mortality rate (9%) was observed in patients receiving prednisone at 60 mg/day, followed by 80 mg/day (10%), and a treatment duration of 14 days was associated with the lowest mortality (9%), see the appendix section in Supplementary Table S5. These findings broadly align with current HIV-positive PCP guidelines (Panel on Opportunistic Infections in Adults and Adolescents with HIV, 2024), which recommend a 21-day glucocorticoid course. However, our sensitivity subgroup analysis showed lower mortality with 14-day treatment, possibly because prolonged glucocorticoid exposure may contribute to increase mortality through immunosuppression. The study populations also varied, ranging from adults with moderate-to-severe PCP to children with HIV infection (Alta et al., 2008; Newberry et al., 2017), in whom drug disposition differs substantially from that achieved in adults. Subgroup analyses also revealed that mortality rates in the glucocorticoid group were higher in children than in adults (32% vs. 11%), see the appendix section in Supplementary Table S5. Such clinical diversity may affect the external validity of the pooled estimates. Thus, the benefits demonstrated in this study should be cautiously extrapolated to high-dose, prolonged-course, and pediatric glucocorticoid use in patients who are HIV-positive with PCP.

This meta-analysis has several limitations that should be acknowledged. (1) Nine articles comprising 13 studies were included. Although the number of high-quality studies was sufficient, the total sample size remained relatively small, which may have increased the probability of false-positive or false-negative results. (2) Most included studies were published earlier and consisted of limited or outdated diagnostic/therapeutic approaches that might have introduced potential bias. (3) Some of the literature derived from multicenter clinical trials (Walmsley et al., 1995), in which study participants originated from diverse locations. Individual differences within the enrolled populations and variations in the severity of underlying diseases may all have contributed to selection bias. (4) Three studies had some concerns regarding bias risk. Although sensitivity analyses sequentially excluding each study showed no effective impact on pooled effect estimates, and GRADE assessment indicated limited influence on the overall rating, the potential selection bias and prescription bias from these trials cannot be completely excluded.

In summary, the treatment regimen containing glucocorticoids demonstrates good clinical efficacy for patients who are HIV-positive with PCP infection. Adjuvant glucocorticoid therapy significantly reduced mortality, early deterioration, and mechanical ventilation rates in patients who are HIV-positive with PCP without increasing adverse reactions. Therefore, the glucocorticoid-containing therapeutic regimen could serve as an important adjunctive treatment option for patients who are HIV-positive with PCP infection.

## 5 Conclusion

Adjuvant glucocorticoid therapy significantly reduces mortality, early deterioration, and mechanical ventilation rates in patients who are HIV-positive with PCP without increasing adverse reactions. Glucocorticoids may serve as a viable option for adjuvant treatment of PCP in patients who are HIV-positive. Future large-scale, multicenter RCTs are warranted to confirm these findings and to better define the optimal glucocorticoid agent, dose, and duration (including shorter tapering regimens), and evaluate efficacy and safety in special populations such as infants. Standardized AE reporting and individual-participant data meta-analyses would further strengthen the evidence base.

## Data Availability

The original contributions presented in the study are included in the article/Supplementary Material, further inquiries can be directed to the corresponding author.
