# Supplementary material for: Efficacy and safety of adjuvant glucocorticoid therapy for Pneumocystis pneumonia (PCP) in patients positive for HIV: a systematic review and meta-analysis
Source: Front Pharmacol. 2026 Oct 28;17:1916907. doi: 10.3389/fphar.2026.1916907 (PMC13752913; doi:10.3389/fphar.2026.1916907)
Supplement: Supplementary file 1 [file Table1.pdf]

Supplementary Table 1. Search strategy.

**PubMed:** 577 records (up to December 10, 2025)<br>(Pneumocystis Pneumonia OR Pneumocystis Infections OR Pneumocystis carinii OR Pneumocystis jirovecii[MeSH Terms]) AND (glucocorticoid OR corticosteroid OR steroid[MeSH Terms]) AND (HIV OR AIDS OR acquired immunodeficiency syndrome OR human immunodeficiency virus[MeSH Terms])
**EMBASE:** 464 records (up to December 10, 2025)<br>('pneumocystis pneumonia':ti,ab,kw OR 'pneumocystis infections':ti,ab,kw OR 'pneumocystis jirovecii':ti,ab,kw OR 'Pneumocystis carinii':ti,ab,kw) AND ('glucocorticoid':ti,ab,kw OR 'corticosteroid':ti,ab,kw OR 'steroid':ti,ab,kw) AND ('hiv':ti,ab,kw OR 'aids':ti,ab,kw OR 'acquired immunodeficiency syndrome':ti,ab,kw OR 'human immunodeficiency virus':ti,ab,kw)
**Web of Science:** 641 records (up to December 10, 2025)<br>TS=("pneumocystis pneumonia" OR "pneumocystis infections" OR "pneumocystis jirovecii" OR "Pneumocystis carinii") AND TS=(glucocorticoid OR corticosteroid OR steroid) AND TS=(hiv OR aids OR "acquired immunodeficiency syndrome" OR "human immunodeficiency virus")
**Cochrane Library:** 30 records (up to December 10, 2025)<br>(Pneumocystis Pneumonia OR Pneumocystis Infections OR Pneumocystis carinii OR Pneumocystis jirovecii):ti,ab,kw AND (glucocorticoid OR corticosteroid OR steroid):ti,ab,kw AND (HIV OR AIDS OR acquired immunodeficiency syndrome OR human immunodeficiency virus):ti,ab,kw
