# Supplementary material for: Efficacy and safety of adjuvant glucocorticoid therapy for Pneumocystis pneumonia (PCP) in patients positive for HIV: a systematic review and meta-analysis
Source: Front Pharmacol. 2026 Oct 28;17:1916907. doi: 10.3389/fphar.2026.1916907 (PMC13752913; doi:10.3389/fphar.2026.1916907)
Supplement: Supplementary file 2 [file Table2.pdf]

Supplementary Table 2. The risk of bias assessment for each included study using the RoB 2 tool.

Study	Randomization process	Deviations from intended interventions	Missing outcome data	Measurement of the outcome	Selection of the reported result	Risk of bias
William 2020	Some concerns	Some concerns	Low risk	Low risk	Low risk	Some concerns
Julio 1989	Some concerns	Some concerns	Low risk	Low risk	Low risk	Some concerns
Samuel 1990	Low risk	Some concerns	Low risk	Low risk	Low risk	Some concerns
Sharon1995	Low risk	Low risk	Low risk	Low risk	Low risk	Low risk
Montaner 1993	Low risk	Low risk	Low risk	Low risk	Low risk	Low risk
Alta 2008	Low risk	Low risk	Low risk	Low risk	Low risk	Low risk
Suzanne 1990	Low risk	Low risk	Low risk	Low risk	Low risk	Low risk
Julio 1990	Low risk	Low risk	Low risk	Low risk	Low risk	Low risk
Laura 2017	Low risk	Low risk	Low risk	Low risk	Low risk	Low risk
