# Supplementary material for: Efficacy and safety of adjuvant glucocorticoid therapy for Pneumocystis pneumonia (PCP) in patients positive for HIV: a systematic review and meta-analysis
Source: Front Pharmacol. 2026 Oct 28;17:1916907. doi: 10.3389/fphar.2026.1916907 (PMC13752913; doi:10.3389/fphar.2026.1916907)
Supplement: Supplementary file 3 [file Table3.pdf]

Supplementary Table 3. Egger's test for mortality rates.

Std_Eff	Coef.	Std. Err.	t	P>|t|	[95% Conf. Interval]	
slope	-0.2686283	0.5097633	-0.53	0.611	-1.421793	0.8845363
bias	-1.116111	0.7908871	-1.41	0.192	-2.905221	0.6730003
