# Supplementary material for: Efficacy and safety of adjuvant glucocorticoid therapy for Pneumocystis pneumonia (PCP) in patients positive for HIV: a systematic review and meta-analysis
Source: Front Pharmacol. 2026 Oct 28;17:1916907. doi: 10.3389/fphar.2026.1916907 (PMC13752913; doi:10.3389/fphar.2026.1916907)
Supplement: Supplementary file 4 [file Table4.pdf]

Supplementary Table 4. Results of heterogeneity analysis based on the mortality rate.

Excluded Study	P	I^2
Laura-a 2017	P＜0.0001	40%
Laura-b 2017	P＜0.0001	39%
Montaner1993	P＜0.00001	33%
Samuel 1990	P＜0.0001	40%
Sharon 1995	P＜0.00001	39%
Suzanne 1990	P＜0.0001	27%
William-a 2020	P＜0.00001	40%
William-b 2020	P＜0.00001	30%
William-c 2020	P＜0.00001	30%
Alta 2008	P＜0.00001	6%
Julio 1989	P＜0.00001	36%
