# Supplementary material for: Efficacy and safety of adjuvant glucocorticoid therapy for Pneumocystis pneumonia (PCP) in patients positive for HIV: a systematic review and meta-analysis
Source: Front Pharmacol. 2026 Oct 28;17:1916907. doi: 10.3389/fphar.2026.1916907 (PMC13752913; doi:10.3389/fphar.2026.1916907)
Supplement: Supplementary file 5 [file Table5.pdf]

Supplementary Table 5. Sensitivity subgroup analysis based on different influencing factors from heterogeneous sources.

	Follow-up duration		Types of glucocorticoids		Dose of glucocorticoids		Treatment duration		Patient population	
Outcomes	P	I^2	P	I^2	P	I^2	P	I^2	P	I^2
Mortality rate	P＜0.00001	34%	P＜0.00001	34%	P＜0.00001	34%	P＜0.00001	34%	P＜0.00001	34%
Early deterioration rate	—	—	—	—	—	—	P=0.002	0%	—	—
Mechanical ventilation rate	P＜0.0001	43%	P＜0.0001	43%	P＜0.0001	43%	P＜0.0001	43%	—	—
Rate of AEs	—	—	P=0.92	59%	P=0.92	59%	—	—	P=0.92	59%

Note: The dash (—) indicates that subgroup comparisons could not be performed because only one subgroup had sufficient data; hence, P and I^2 are not shown.

Appendix:

（1）Sensitivity subgroup analyses stratified by follow-up duration

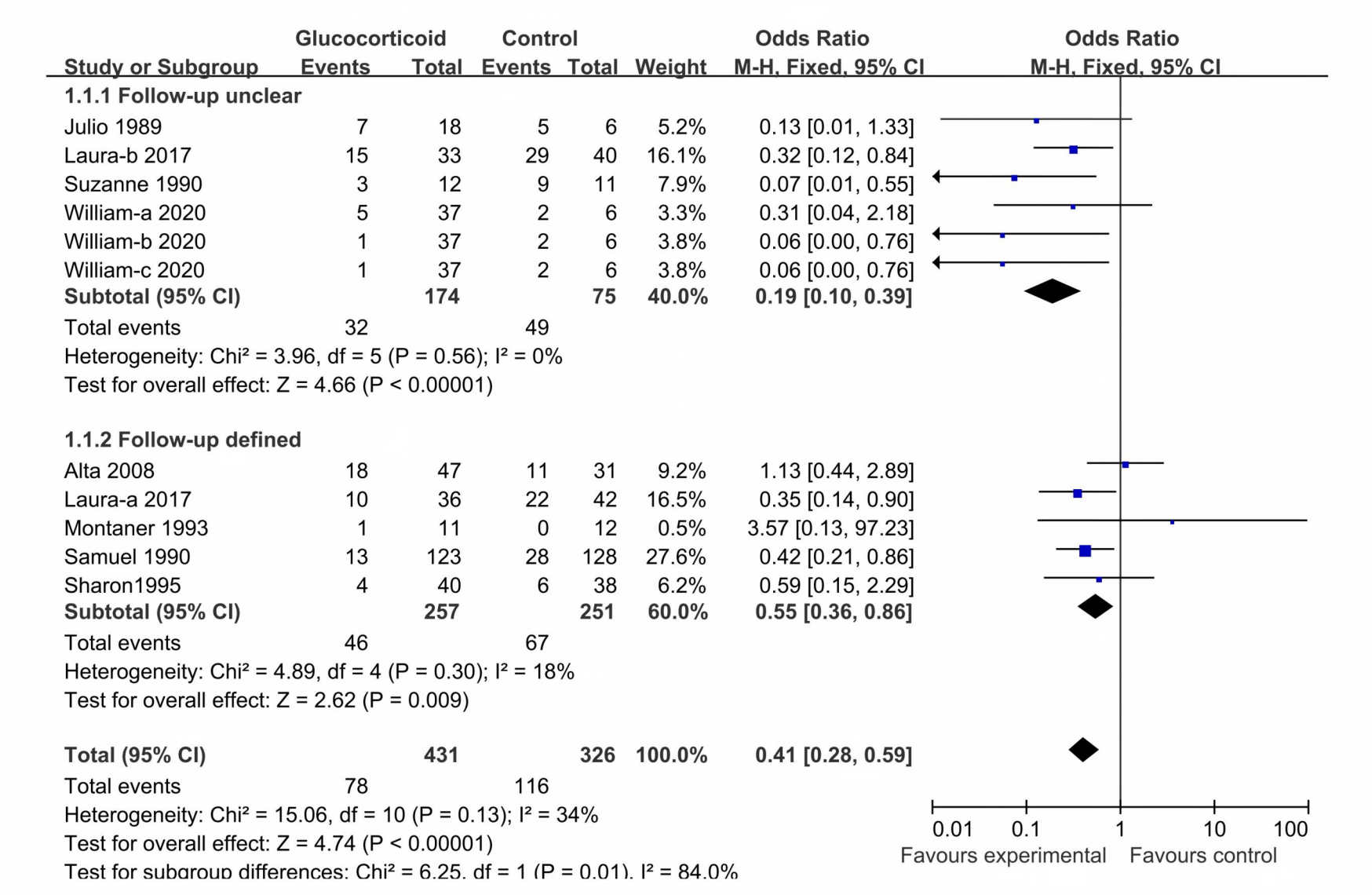

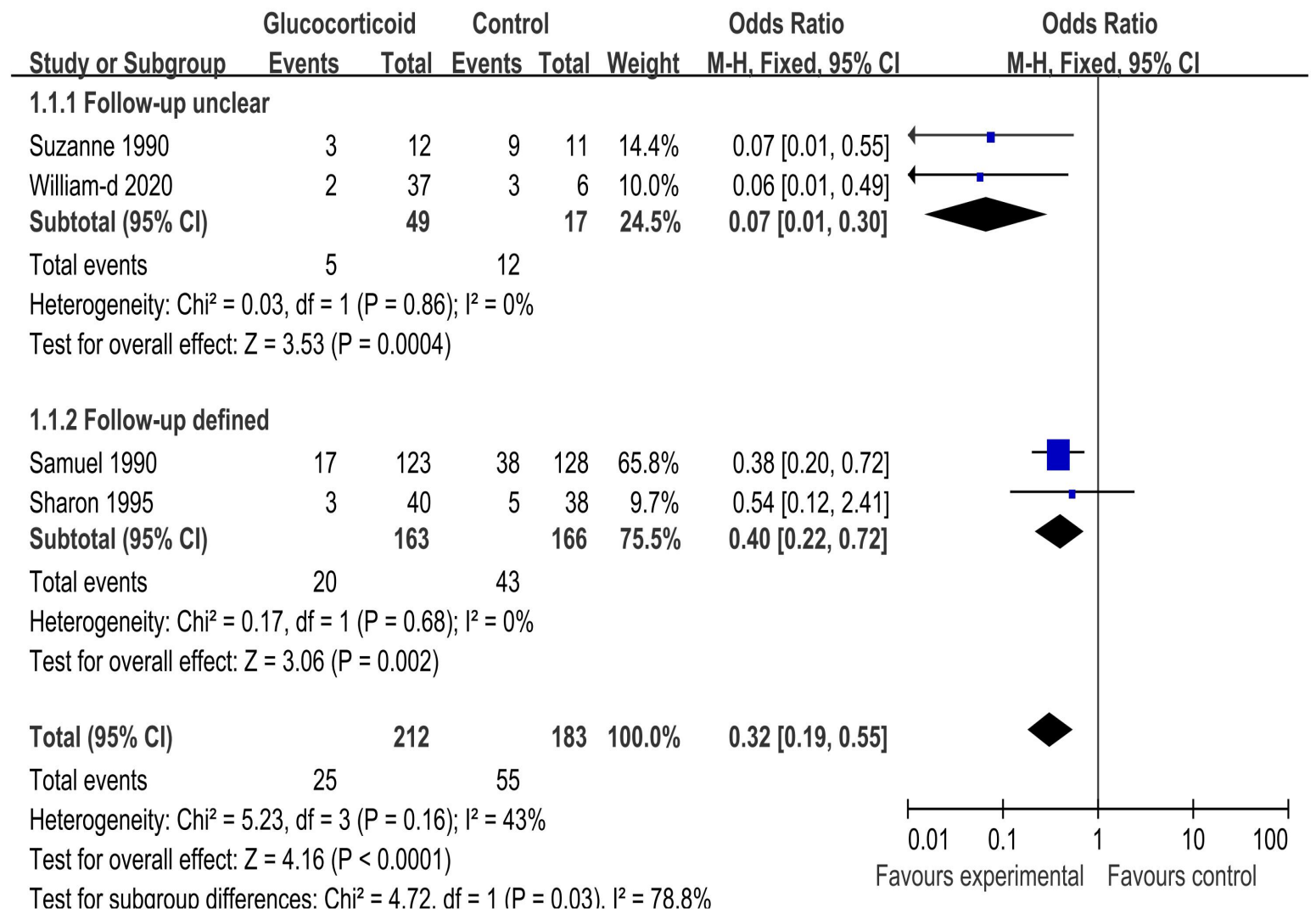

Forest plot of the rate of mortality

Forest plot of the rate of mechanical ventilation

（2）Sensitivity subgroup analyses stratified by glucocorticoids type

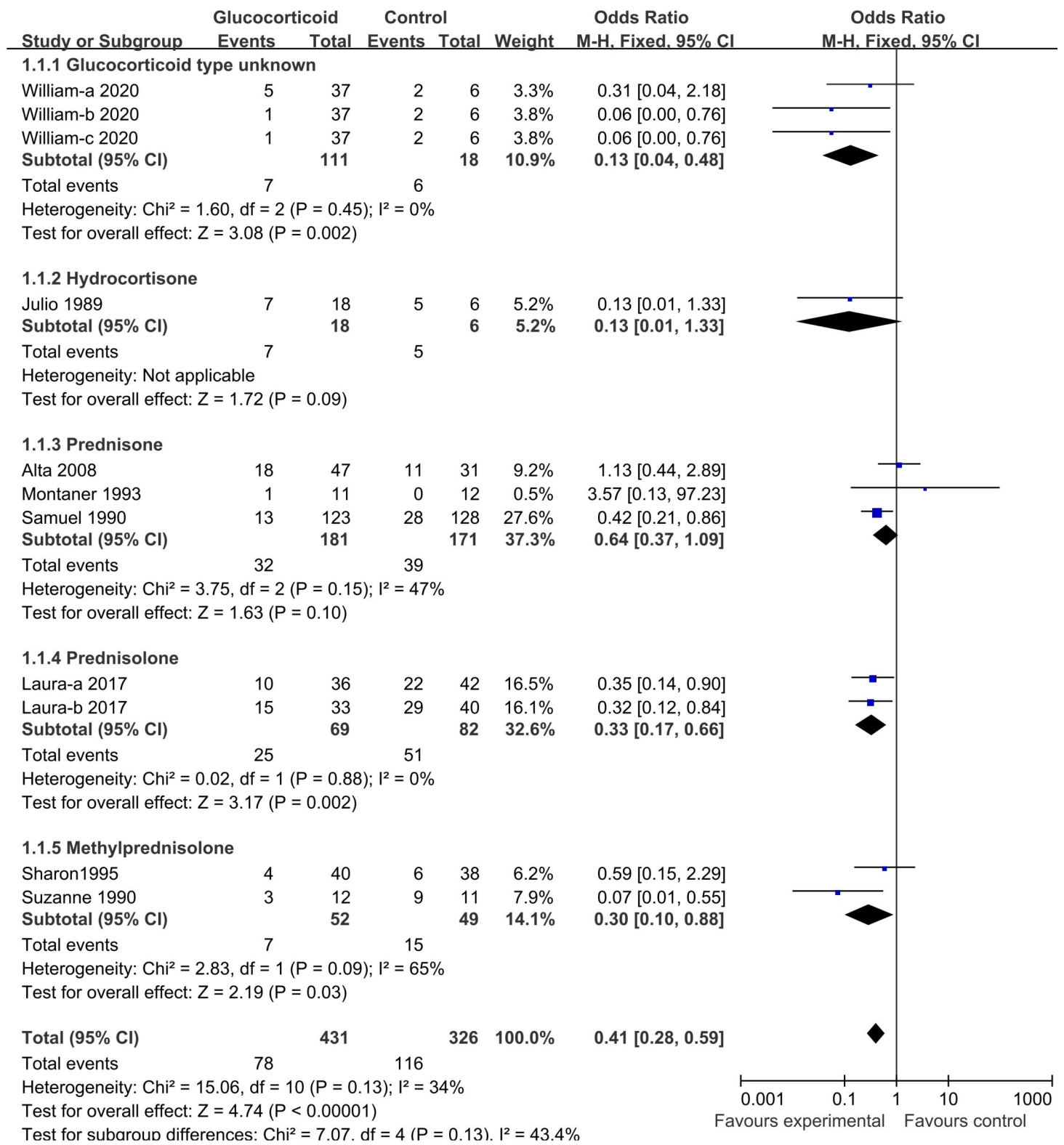

Forest plot of the rate of mortality

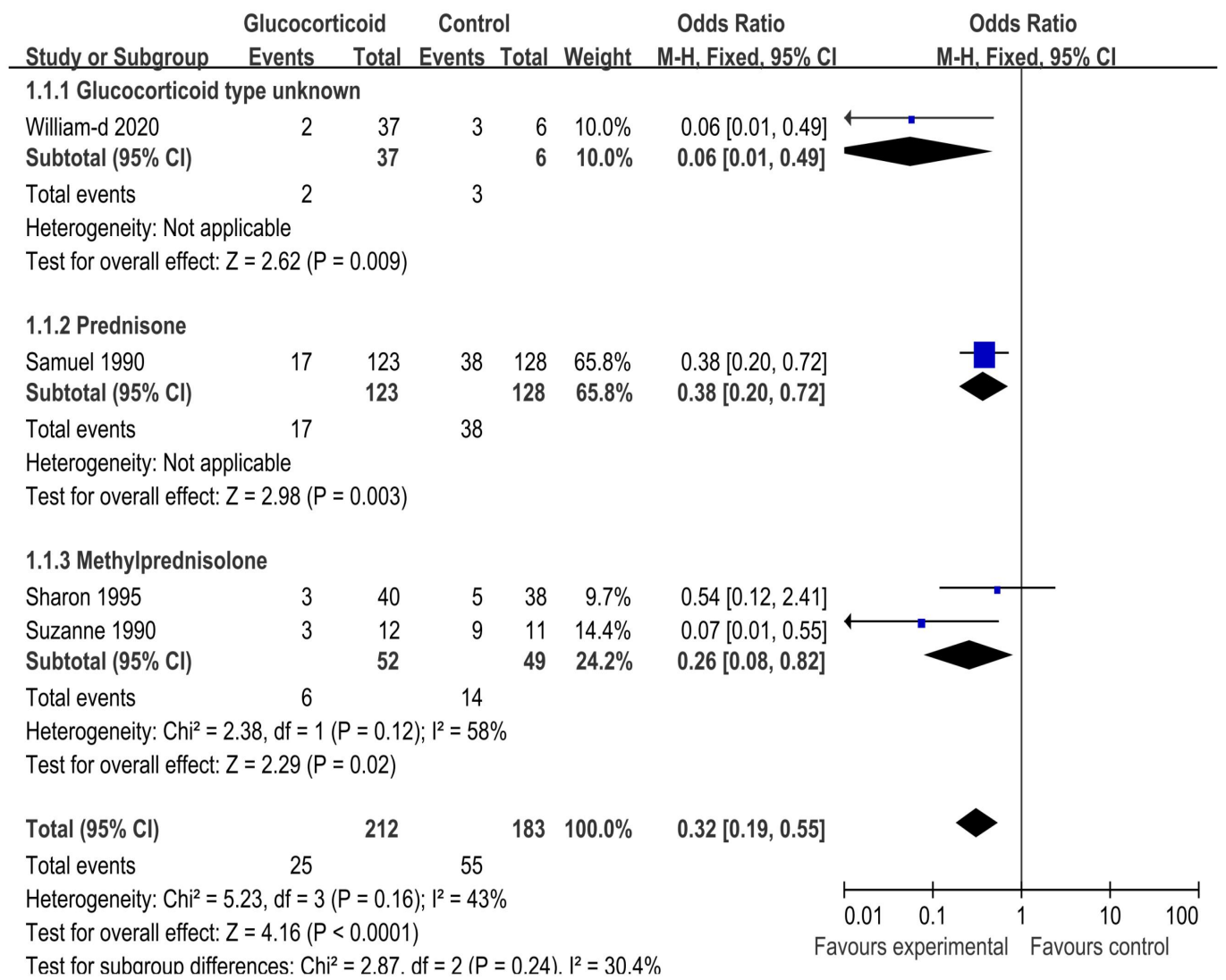

Forest plot of the rate of mechanical ventilation

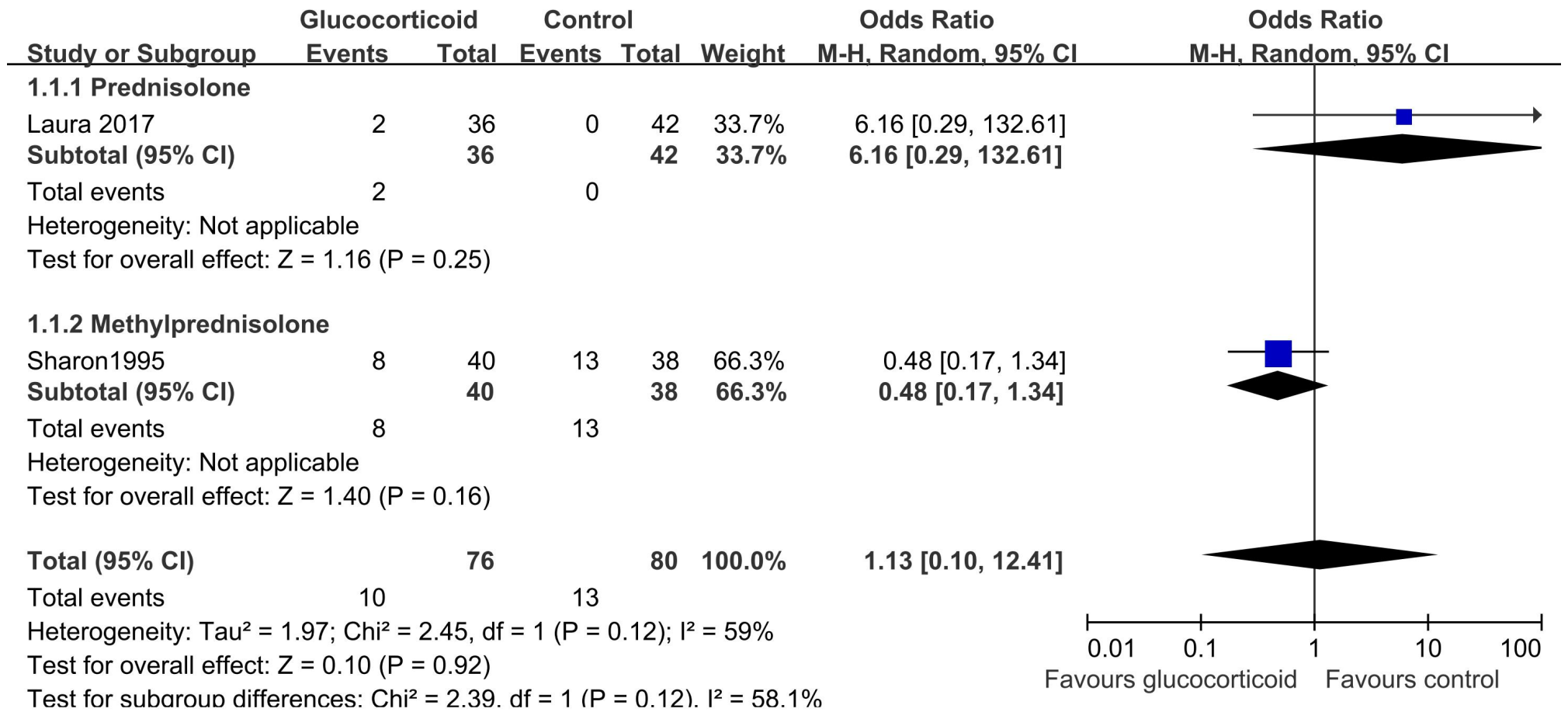

Forest plot of the rate of AEs

（3）Sensitivity subgroup analyses stratified by glucocorticoid dose

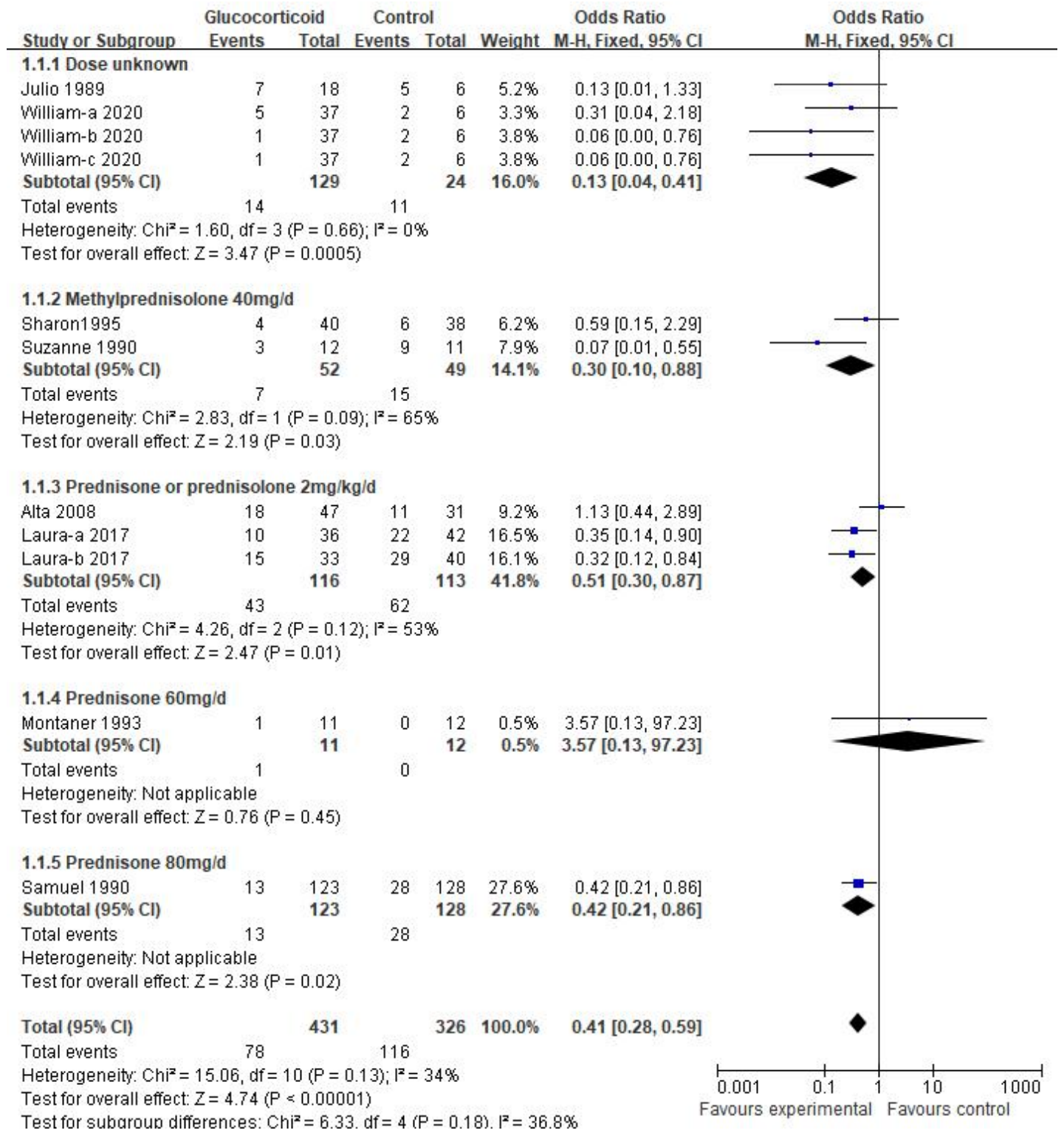

Forest plot of the rate of mortality

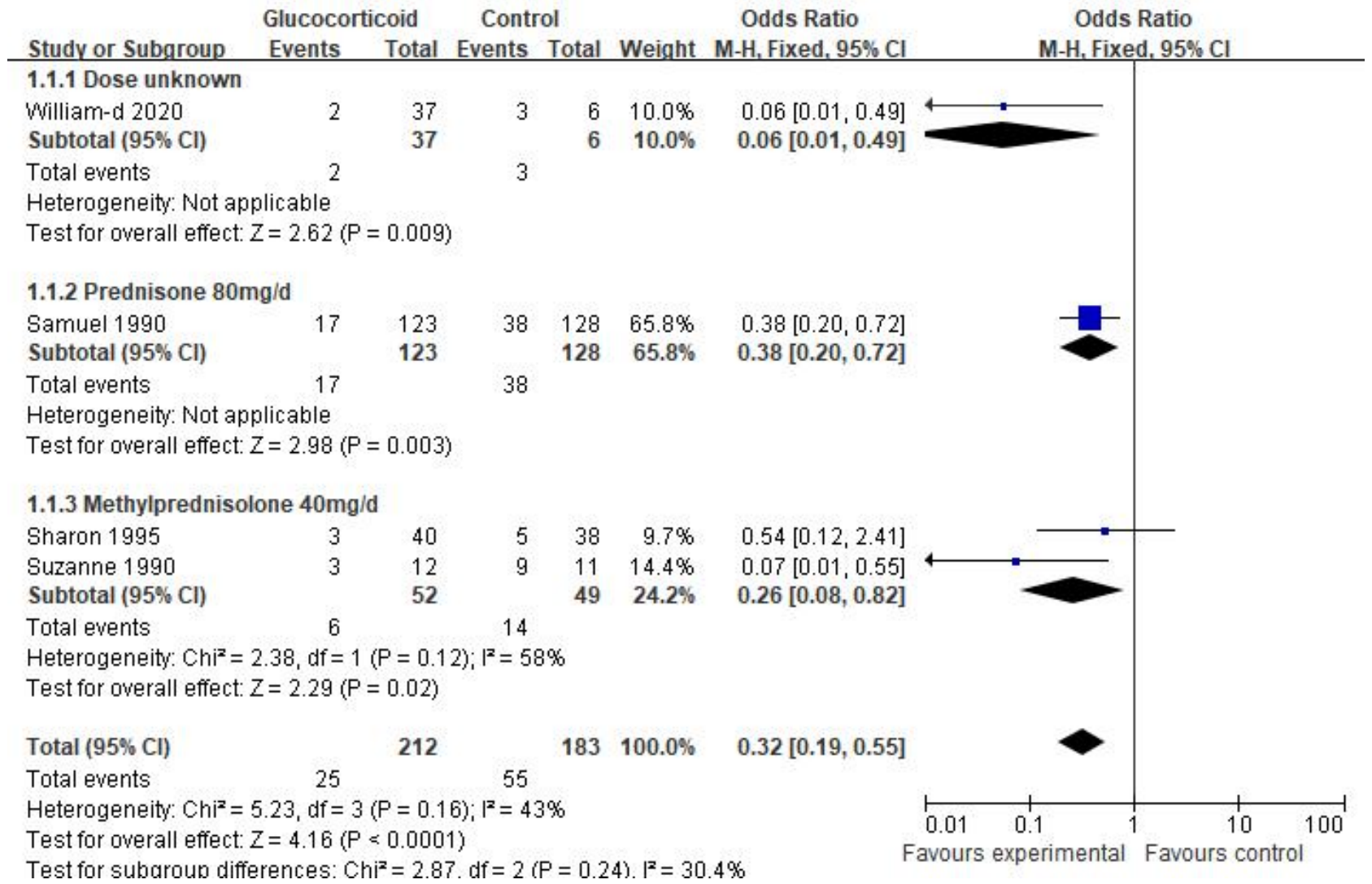

Forest plot of the rate of mechanical ventilation

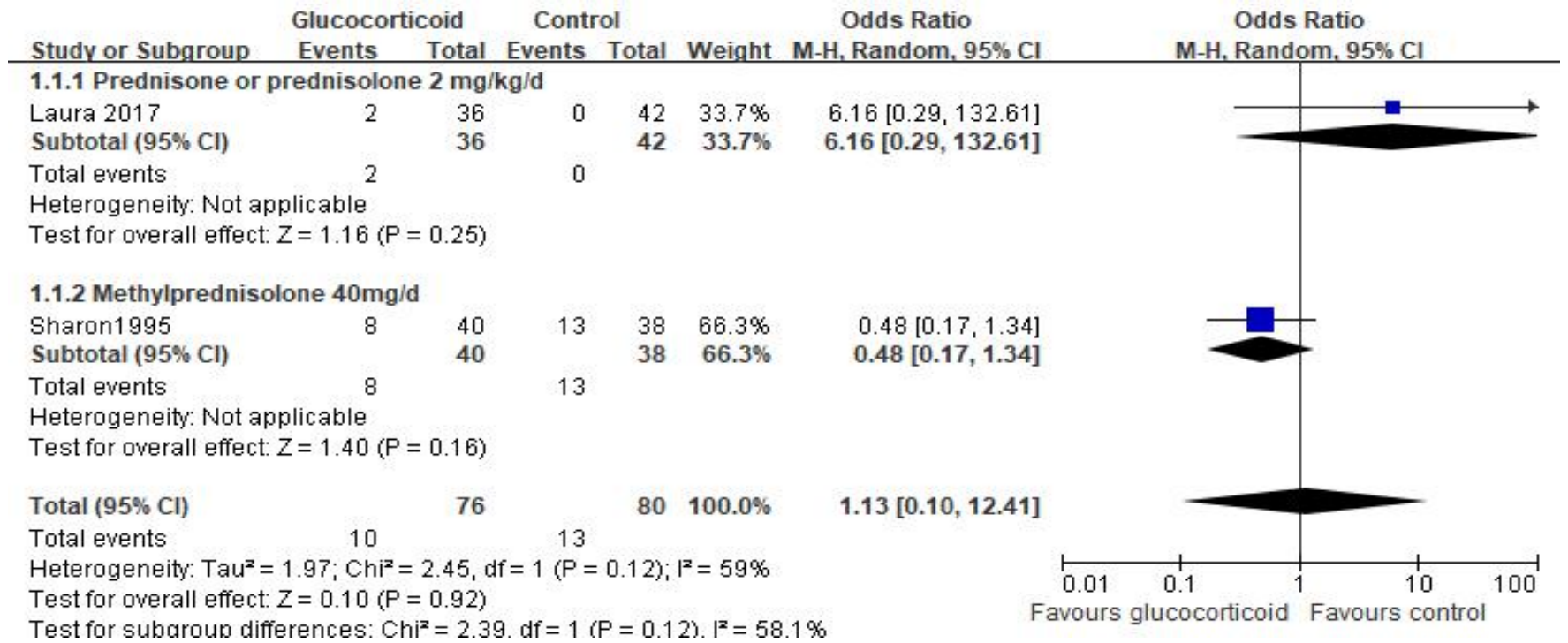

Forest plot of the rate of AEs

（4）Sensitivity subgroup analyses stratified by treatment duration

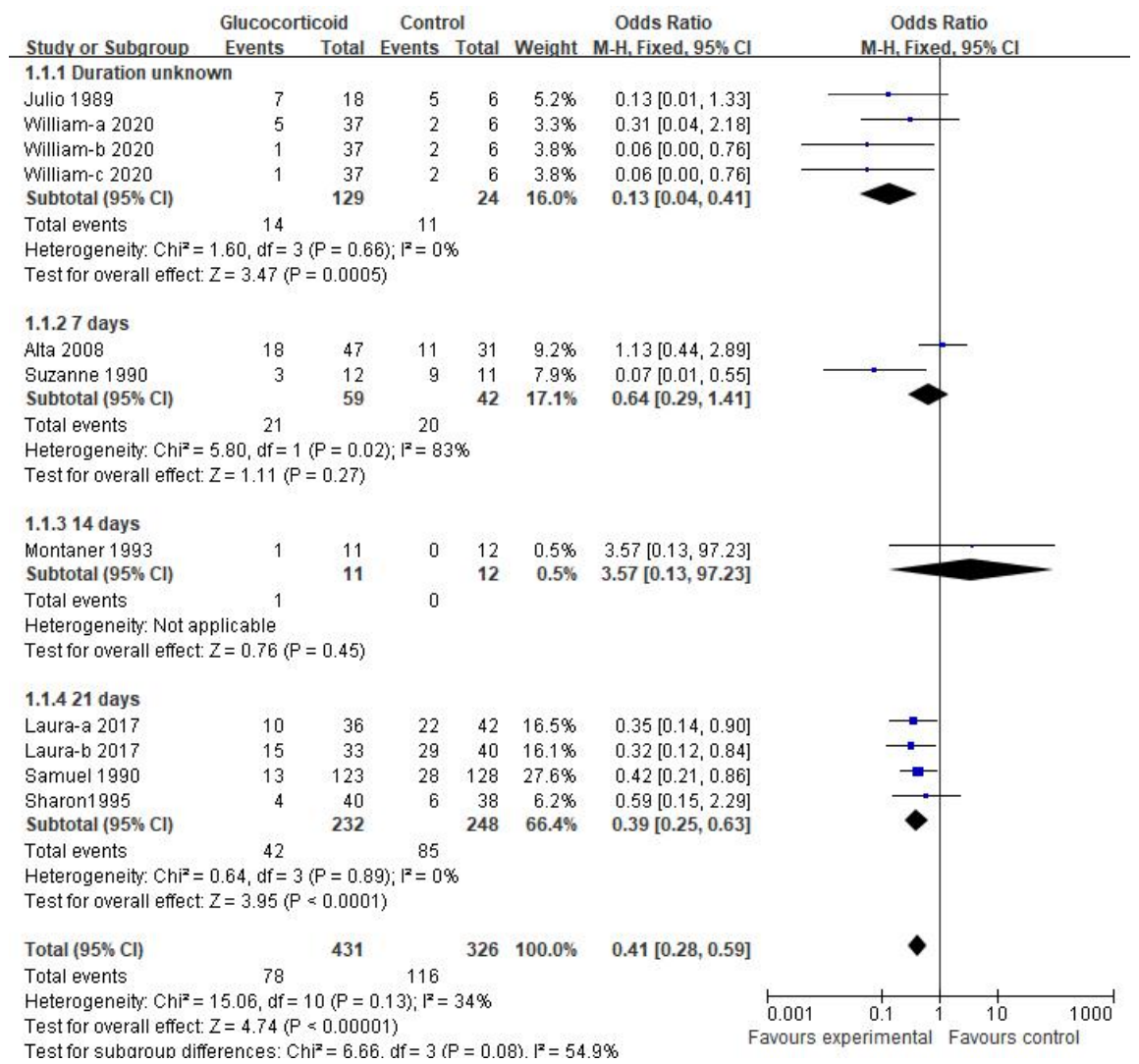

Forest plot of the rate of mortality

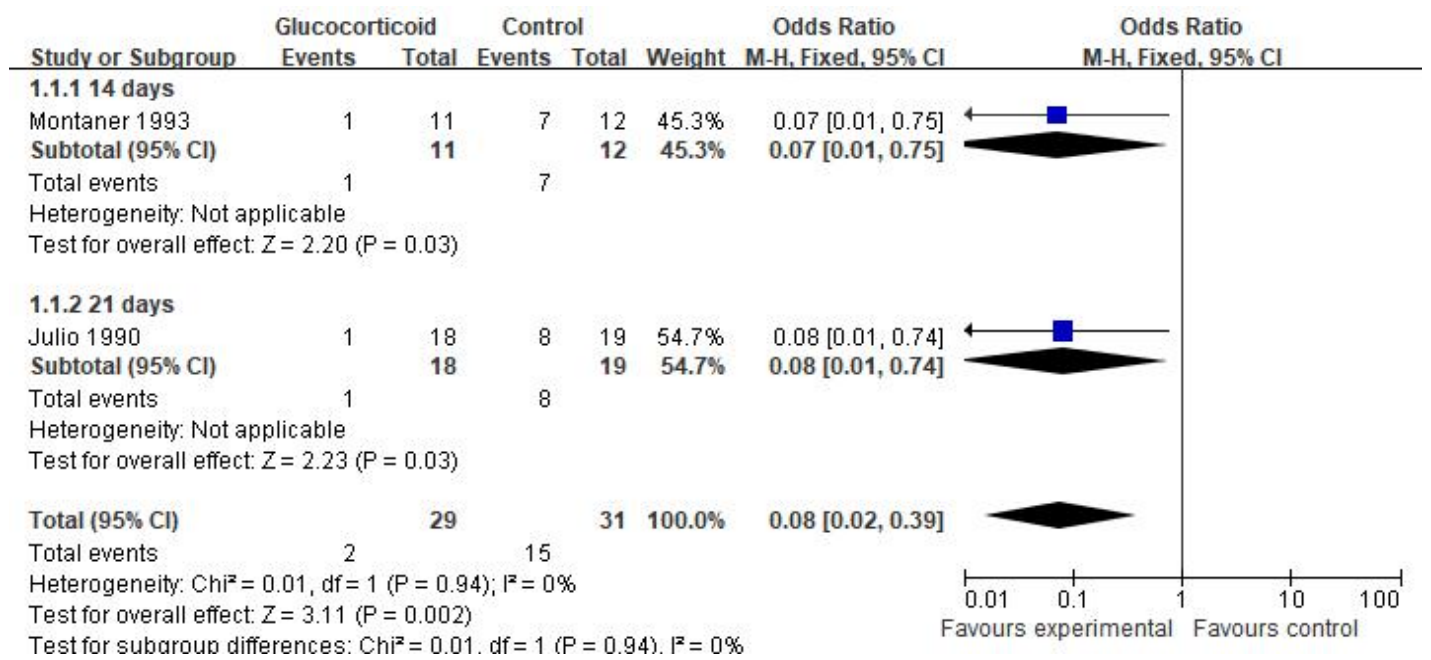

Forest plot of the rate of early deterioration

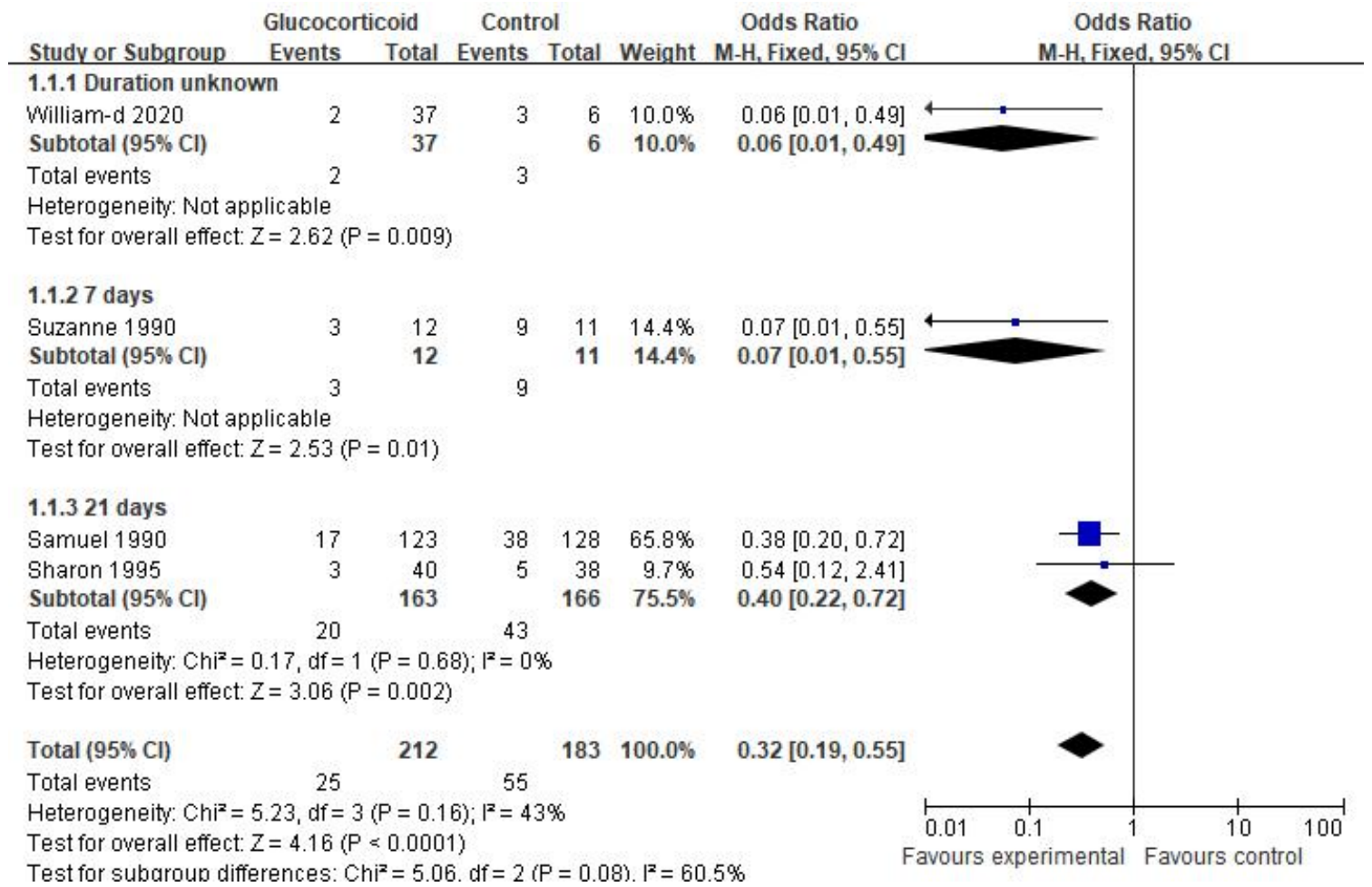

Forest plot of the rate of mechanical ventilation

（5）Sensitivity subgroup analyses stratified by patient population

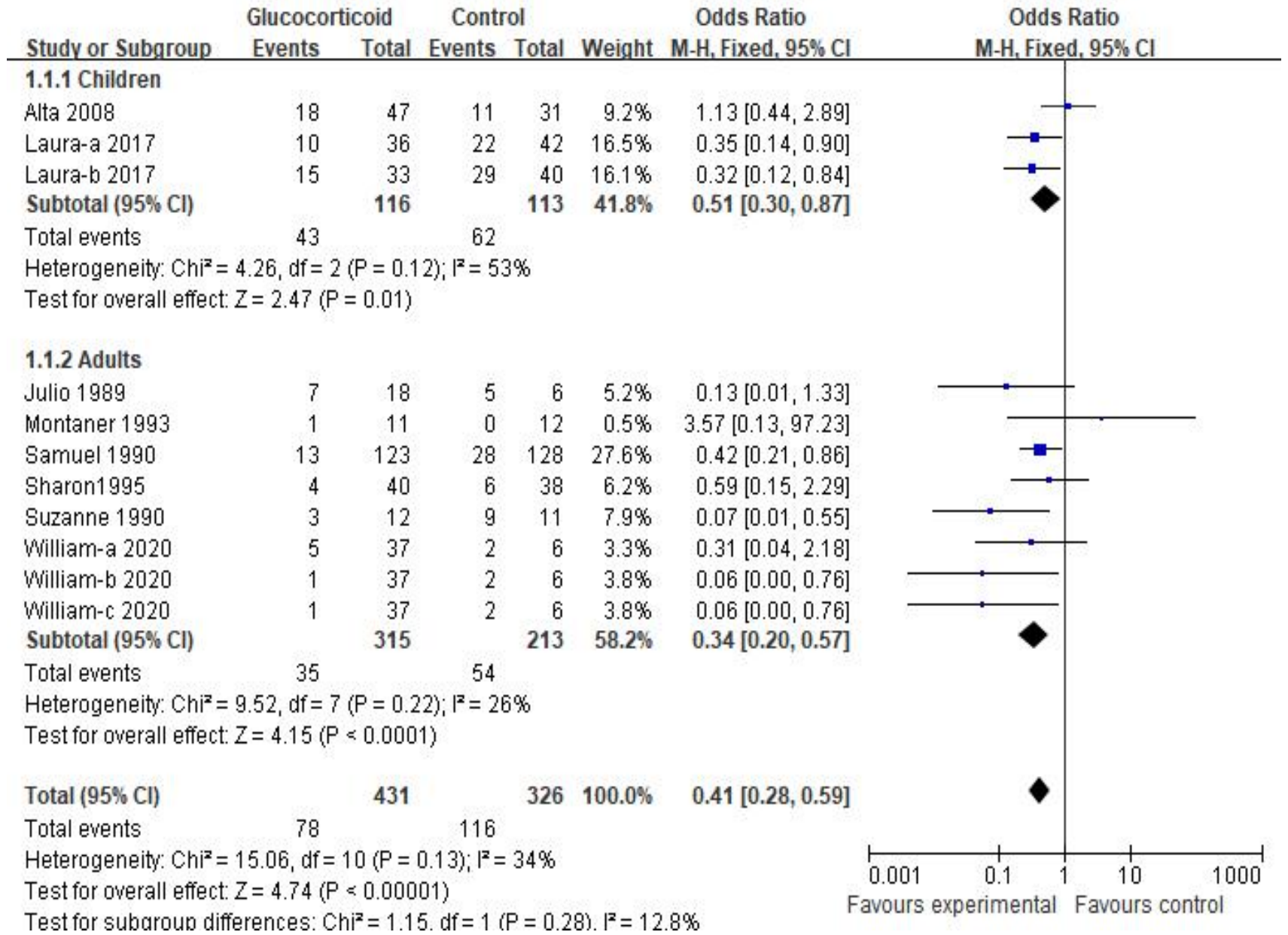

Forest plot of the rate of mortality

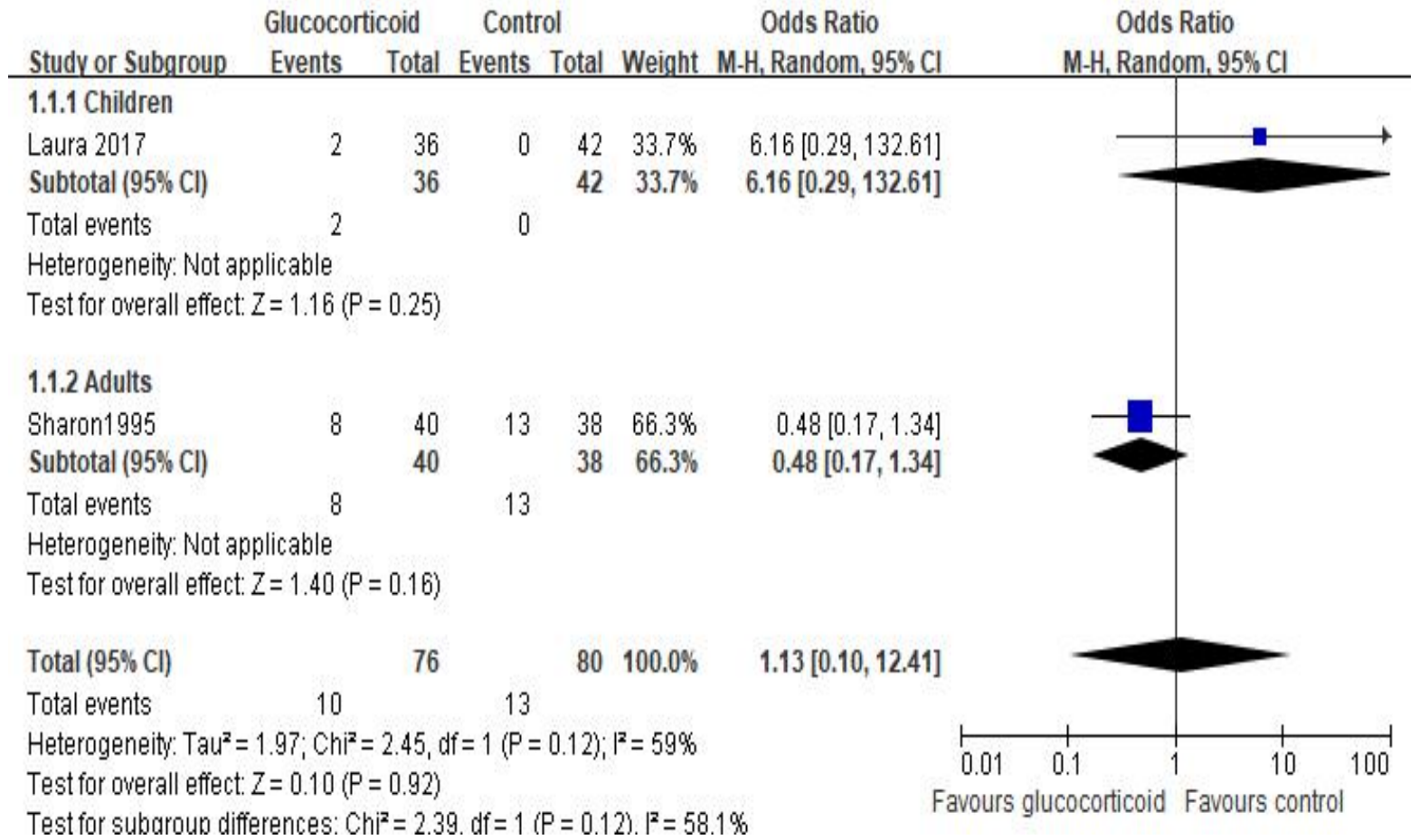

Forest plot of the rate of AEs
