# Supplementary material for: Efficacy and safety of adjuvant glucocorticoid therapy for Pneumocystis pneumonia (PCP) in patients positive for HIV: a systematic review and meta-analysis
Source: Front Pharmacol. 2026 Oct 28;17:1916907. doi: 10.3389/fphar.2026.1916907 (PMC13752913; doi:10.3389/fphar.2026.1916907)
Supplement: Supplementary file 6 [file Table6.pdf]

Supplementary Table 6. GRADE evidence profile for adjuvant glucocorticoid therapy in HIV-positive patients with Pneumocystis pneumonia (PCP)

Outcome	No. of studies (design)	Risk of bias	Inconsistency	Indirectness	Imprecision	Publication bias	No. of patients (GC / Control)	Relative effect OR (95% CI)	Certainty (GRADE)	Importance
**Mortality rate**	11 studies from 8 articles (9 RCTs, 2 CCTs)	Not serious[a]	Not serious (I^2 = 34%, P = 0.13)	Not serious[b]	Not serious	Undetected[d]	431 / 326	0.41 (0.28–0.59)	⊕ ⊕ ⊕ ⊕ High	Critical
**Early deterioration rate**	2 studies (2 RCTs)	Not serious	Not serious (I^2 = 0%, P = 0.94)	Not serious[b]	Serious[c]	Not assessable[e]	29 / 31	0.08 (0.02–0.39)	⊕ ⊕ ⊕ ⊝ Moderate	Critical
**Mechanical ventilation rate**	4 studies (3 RCTs, 1 CCT)	Not serious[a]	Not serious (I^2 = 43%, P = 0.16)	Not serious[b]	Not serious	Not assessable[e]	212 / 183	0.32 (0.19–0.55)	⊕ ⊕ ⊕ ⊕ High	Critical
**Incidence of adverse events (AEs)**	2 studies (2 RCTs)	Not serious	Not serious (I^2 = 29%, P = 0.23)	Not serious	Serious[c]	Not assessable[e]	78 / 80	0.74 (0.33–1.70)	⊕ ⊕ ⊕ ⊝ Moderate	Critical

Abbreviations: RCT, randomized controlled trial; CCT, clinical controlled trial; GC, glucocorticoid; OR, odds ratio; CI, confidence interval; AEs, adverse events.

GRADE Working Group grades of evidence: High certainty (⊕ ⊕ ⊕ ⊕): very confident that the true effect lies close to that of the estimate of the effect; Moderate certainty (⊕ ⊕ ⊕ ⊝): moderately confident in the effect estimate—the true effect is likely to be close to the estimate, but there is a possibility that it is substantially different; Low certainty (⊕ ⊕ ⊝ ⊝): confidence in the effect estimate is limited—the true effect may be substantially different from the estimate; Very low certainty (⊕ ⊝ ⊝ ⊝): very little confidence in the effect estimate—the true effect is likely to be substantially different from the estimate.

a Not downgraded for risk of bias: although three included articles were judged as having an unclear risk of bias, sensitivity analyses that sequentially excluded each study showed that these trials had no material impact on the pooled effect estimates, with the direction and magnitude of the effects remaining unchanged (S4 Table).

b Not downgraded for indirectness despite clinical diversity (trials spanning 1989–2020; different glucocorticoid agents, doses, routes, and populations including infants), because subgroup and sensitivity analyses stratified by follow-up duration, types of glucocorticoids, dose of glucocorticoids, treatment duration and patient population yielded consistent results (S5 Table).

c Downgraded one level for imprecision: only two small trials were available, with a limited total sample size, few outcome events, and the optimal information size not being met. The evidence was downgraded by only one level because the results were consistent across the contributing trials (I^2 = 0% and 29%, respectively) and the effect estimates were stable.

d Egger's test for the mortality outcome showed no statistically significant publication bias (P = 0.192; S3 Table).

e Publication bias could not be formally assessed because fewer than 10 studies were available for funnel-plot/Egger's test analyses.
